# A case of pleural and pericardium amyloidosis with effusion associated with multiple myeloma relapse

**DOI:** 10.1002/rcr2.1445

**Published:** 2024-07-31

**Authors:** Naoya Ishibashi, Takafumi Sugawara, Yugo Ashino, Ryuga Yabe, Ryo Nonomura, Yutaka Oshima, Takanobu Sasaki

**Affiliations:** ^1^ Department of Thoracic Surgery Tohoku Medical and Pharmaceutical University Hospital Sendai‐shi Japan; ^2^ Department of Respiratory Medicine Sendai City Hospital Sendai‐shi Japan

**Keywords:** amyloidosis, multiple myeloma, pleural effusion

## Abstract

An 80‐year‐old man with a history of Bence‐Jones potein (BJP) λ‐type multiple myeloma (MM), which had been in remission for 16 years, was examined for shortness of breath and was found to have bilateral pleural and pericardial effusions. A pleural fluid test and a pleural biopsy under local anaesthesia performed by a previous physician failed to make the diagnosis. Despite diuretic therapy, his condition necessitated frequent thoracentesis. The patient was referred to our hospital and thoracoscopic pleural and pericardial biopsies performed under general anaesthesia revealed λ‐type AL amyloidosis, indicating a relapse of MM. Despite drug therapy for MM, the patient died from aspiration pneumonia. The case underscores the importance of considering amyloidosis in differential diagnoses for refractory effusions, especially in patients with a history of MM, even after long‐term remission.

## Graphical abstract

An 80‐year‐old man with a history of multiple myeloma (MM), which had been in remission for 16 years, presented with bilateral pleural and pericardial effusions. Various investigations, including pleural biopsy and imaging studies, led to the patient's diagnosis of lambda AL amyloidosis complicated by relapsed multiple myeloma.

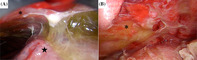

## INTRODUCTION

Amyloidosis is found in 10%–15% of patients with multiple myeloma (MM).^1^ Amyloidosis is an intractable disease in which insoluble amyloid fibrils are deposited in organs throughout the body, causing organ damage. In the present case, Bence‐Jones potein (BJP) λ‐type MM had been in remission for 16 years, but the patient developed refractory pleural effusion. There was no evidence of cardiac or renal involvement, and biopsies of the pleura and pericardium identified amyloidosis.

## CASE REPORT

An 80‐year‐old male patient with a chief complaint of shortness of breath visited his previous physician, and bilateral pleural effusion and pericardial effusion were noted on chest computed tomography (CT). The patient had been previously diagnosed with BJP λ‐type MM in his 60's, stageIIIA. He received vincristine and doxorubicin plus dexamethasone (VAD) treatment, high‐dose etoposide treatment, autologous peripheral blood stem cell transplantation (PBSCT), and a combination of etoposide, dexamethasone, cytarabine and cisplatin. He was subsequently declared in remission and was discharged from follow‐up. Other medical history included atrial fibrillation, for which he was taking edoxaban. There were no significant findings in the family history. Approximately 6 months after his presentation with pleural effusions, five thoracentesis procedures under local anaesthesia and one pleural biopsy had been performed by the previous doctor, but the diagnosis was inconclusive and the pleural effusion became difficult to control, so the patient was referred to our department for comprehensive examination.

**FIGURE 1:**
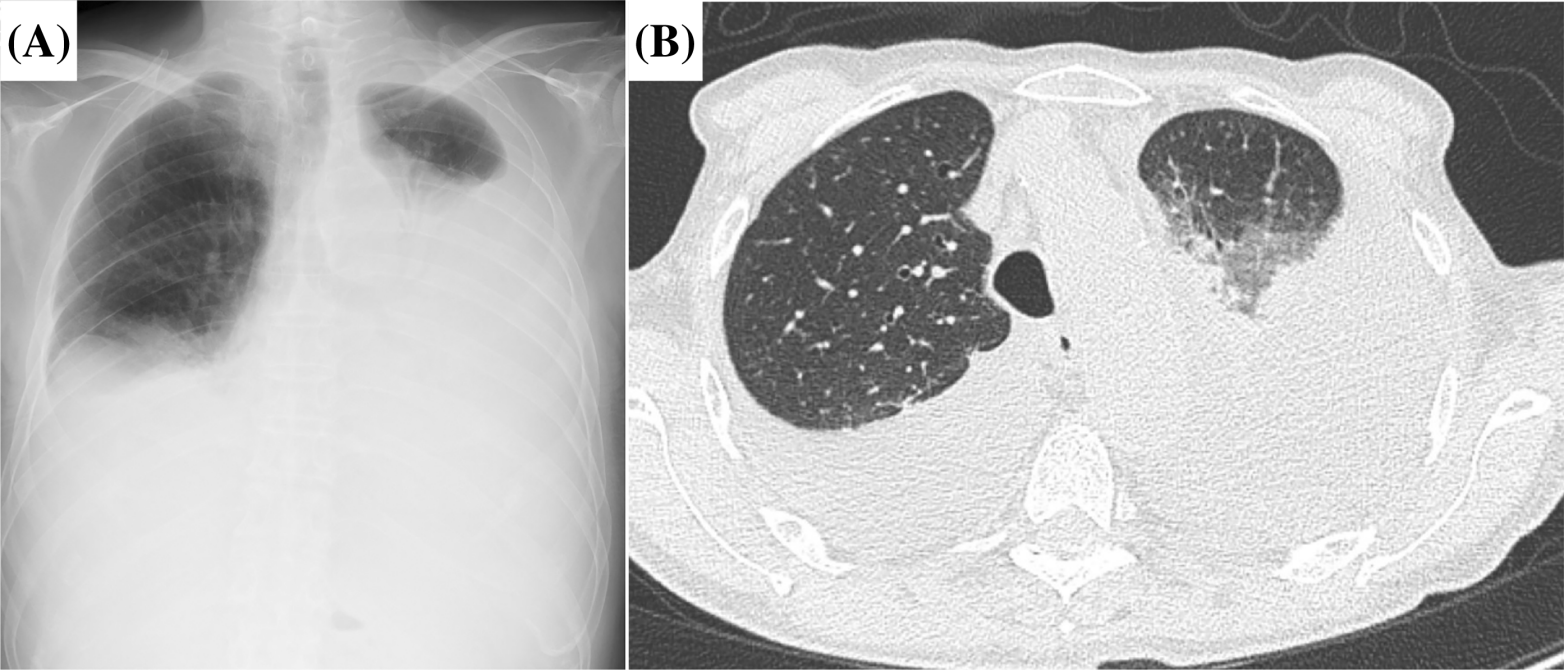
Chest x‐ray on admission showing bilateral moderate to large pleural effusion (A). Chest computed tomography showing collapsed lungs with significant bilateral intrathoracic fluid accumulation (B).

At the time of referral to our hospital, a chest x‐ray showed a moderate to large pleural effusion (Figure 1A). Positron emission tomography (PET)‐CT showed no obvious 18F‐fluorodeoxyglucose (FDG) accumulation in the thoracic cavity. The results of blood sampling showed that the serum total protein was 5.9 g/dL and serum LDH was 168 U/L. Serum immunoelectrophoresis showed no obvious M protein, and urine immunoelectrophoresis showed no obvious Bence Jones protein. Chest CT revealed large amounts of pleural effusions in the bilateral pleural cavities, and a drain was placed in the left pleural cavity to alleviate dyspnea (Figure 1B). The pleural fluid was clear yellow, the total protein in the pleural fluid was 3.8 g/dL, and LDH was 74 U/L, which was an exudative pleural fluid by Light's criteria. A few days later, a drain was placed in the right pleural cavity due to an increased right‐sided pleural effusion. Total protein in the right pleural fluid was 2.9 g/dL and LDH was 60 U/L, indicating a transudative pleural effusion. Both cytology and cell block examination showed no malignancys in the bilateral pleural effusions. Echocardiography showed no significant systolic or diastolic dysfunction.

**FIGURE 2:**
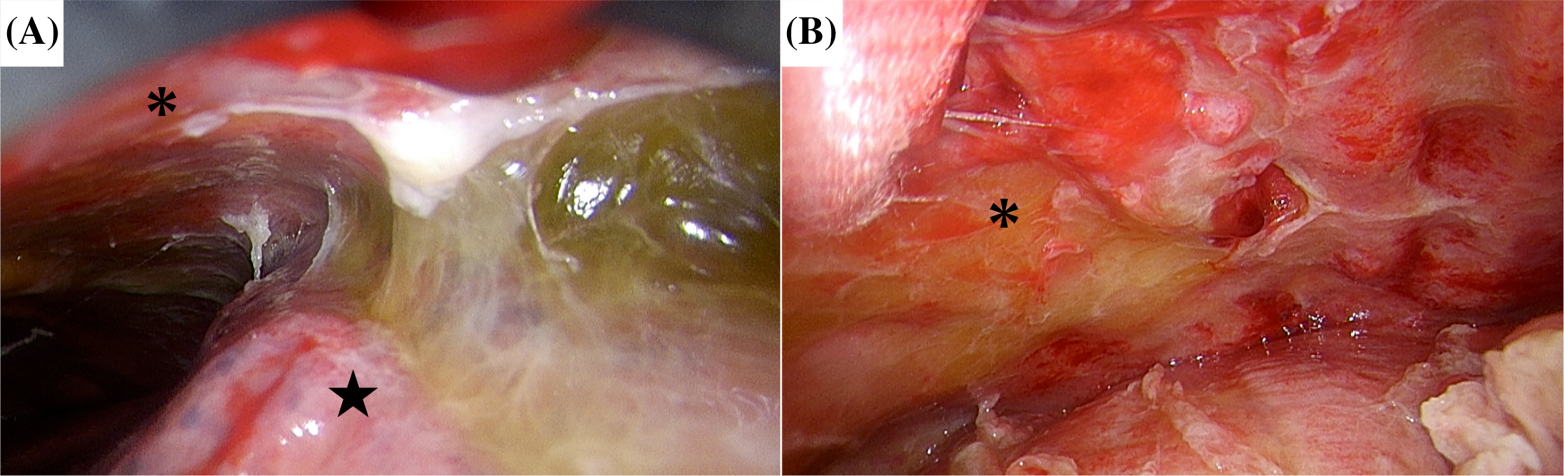
Intraoperative findings. Extensive adhesions between the chest wall (asterisk) and lung (star) (A). The chest wall (asterisk) appeared erythematous and edematous (B).

**FIGURE 3:**
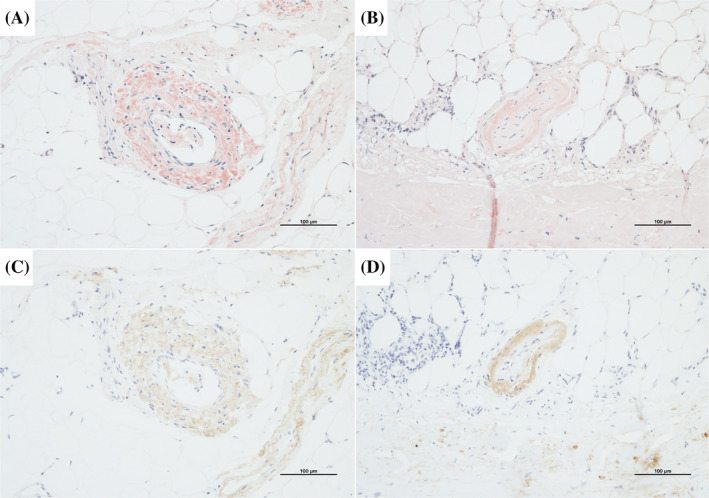
Immunopathological examination. Congo‐red staining showing amyloid deposits in the parietal pleural (A) and pericardial (B) vascular walls. Anti‐lambda antibody staining indicating positive immunoreactivity in the parietal pleura (C) and pericardial (D) vascular walls.

A left thoracoscopic pleural biopsy was suggested to the patient to investigate the cause of the pleural effusion, and the patient agreed. The thoracic cavity was adherent in places, the wall pleura and pericardium were thickened and yellowish‐white to red in colour, and these areas were biopsied (Figure 2). Since the patient was still draining 200–300 mL/day from bilateral thoracic drains after surgery, minocycline 200 mg was administered in the left pleural cavity as a pleurodesis. However, drainage was uncontrolled, and the drains could not be removed. Biopsy specimens of the pleura and pericardium showed anti‐lambda antibody‐positive cells in the same area as the Congo red staining of the amyloid deposits, leading to the diagnosis of lambda‐type AL amyloidosis (Figure 3A,B). His MM, which had been in remission for 16 years, was of the lambda type, therefore he was suspected of having secondary amyloidosis with relapse and was transferred to the haematology department of the referring hospital. A haematologist performed a bone marrow examination and diagnosed MM relapse. Despite treatment with melphalan and prednisolone, he developed liver damage (Grade 3) and was treated with bortezomib as a secondary therapy. However, he subsequently developed aspiration pneumonia and died.

## DISCUSSION

Pleural effusions associated with amyloidosis may be due to cardiac dysfunction caused by cardiac amyloidosis, but the frequency of non‐cardiogenic pleural effusions, such as inhibited reabsorption due to accumulation of amyloid in the pleura, is reported to be 6%.^2^ It is bilateral in55%, with equal proportions of transudative and exudative (43.4% and 42.6%) and reported to be transudative (43.4% and 42.6%).^3^ This patient had no cardiac or renal insufficiency despite bilateral pleural effusions, the right side was a transudative pleural effusion and the left side was an exudative pleural effusion. The cause of the difference in the properties of the left and right pleural effusion is unknown. Theoretically, the protein content of the effusion may have been modified by a number of factors, including diuretics administered by a previous physician prior to referral to our department, local inflammation due to five thoracenteses (two on the right and three on the left, most recently on the left), differences between the right and left pleural lesions, and cardiac diastolic dysfunction due to pericardial damage. The management of refractory pleural effusions differs according to their aetiology. Exudative pleural effusions are caused by fluid accumulation in the pleural cavity due to increased capillary permeability and are often associated with diseases such as pneumonia, pleurisy and lung cancer. If the pleural effusion is due to an inflammatory process, infection control measures should be implemented. On the other hand, if the effusion is due to malignant disease, treatment modalities such as chemotherapy and pleurodesis should be considered.

Congo red staining is useful for diagnosing amyloidosis and it has been reported that pleural fluid cytology may also be effective in diagnosing amyloidosis,^4^ but we decided to pleural biopsy under general anaesthesia for diagnosis. In the case of an unexplained pleural effusion, as many tissue samples as possible should be obtained and Congo Red staining should be considered where the differential diagnosis includes amyloidosis.

Patients with MM complicated by amyloidosis are treated with drugs for MM but may be difficult to treat with potent anticancer drugs due to organ dysfunction caused by amyloid protein. In one study, patients with primary amyloidosis who had pleural involvement, primarily pleural effusions, had a better prognosis in the melphalan plus autologous peripheral blood stem cell transplant group compared to the conventional oral chemotherapy group (21 months vs. 8.8 months).^2^ MP (melphalan, dexamethasone) and CP (cyclophosphamide, prednisone) are standard for patients over 65 years of age or who cannot undergo autologous cell transplantation due to complications. MP therapy was selected for our patient due to his advanced age and poor general condition. The feasibility of treatment should be considered based on the patient's age and organ function. Although MM is difficult to cure completely, the prognosis has improved with the development of various treatment modalities, and the 6‐year survival rate has increased from 31% to 56%, especially in patients over 65 years of age.^5^ On the other hand, pleural amyloidosis, a rare complication of MM, can occur even 16 years after remission and should be included in the differential diagnosis in cases of unexplained pleural effusion.

## AUTHOR CONTRIBUTIONS

Yugo Ashino designed the study; Naoya Ishibashi drafted and edited the manuscript; Takafumi Sugawara critically reviewed the manuscript for important intellectual content. All authors read and revised all drafts of this manuscript and approved the final version of the manuscript.

## CONFLICT OF INTEREST STATEMENT

None declared.

## ETHICS STATEMENT

The authors declare that appropriate written informed consent was obtained for the publication of this manuscript and accompanying images.

## Data Availability

Data available on request due to privacy/ethical restrictions.
